# Antistunting potential of deepsea water concentrate as multivitamin granules: stability and in vivo activity study

**DOI:** 10.1038/s41598-025-21661-8

**Published:** 2025-10-29

**Authors:** Barmi Hartesi, Bambang Retnoaji, Sriwidodo Sriwidodo, Yoga Windhu Wardhana, Muhaimin Muhaimin, Mayang Kusuma Dewi, Sjaikhurrizal El Muttaqien, Anis Yohana Chaerunisaa

**Affiliations:** 1 Doctoral Program, Faculty of Pharmacy, Padjadjaran University, Sumedang, 45363 Indonesia; 2 Department of Tropical Biology, Faculty of Biology, Gadjah Mada University, Yogyakarta, 55281 Indonesia; 3 Department of Pharmaceutics and Pharmaceutical Technology, Faculty of Pharmacy, Padjadjaran University, Sumedang, 45363 Indonesia; 4 Department of Pharmaceutical Biology, Faculty of Pharmacy, Padjadjaran University, Sumedang, 45363 Indonesia; 5 Research Center for Vaccine and Drugs, National Research and Innovation Agency (BRIN), LAPTIAB 1, PUSPIPTEK, South Tangerang, 15314 Indonesia; 6 Central Laboratory, Padjadjaran University, Sumedang, 45363 Indonesia; 7 Department of Pharmaceutics and Pharmaceutical Technology, Faculty of Pharmacy, Jenderal Achmad Yani University, Cimahi, 40531 Indonesia

**Keywords:** Deep seawater, Granule, Hygroscopic, Minerals, Multivitamins, stunting, Biological techniques, Biotechnology, Materials science

## Abstract

Stunting in children is caused by severely inadequate nutrients; therefore, balanced nutritious foods such as multivitamins is the most promising alternative interventions. Concentrated deep seawater is rich in minerals, thus provide potent multivitamin sources. The hygroscopic nature of minerals makes them prone to moisture. Consequently, the fabrication of a non-hygroscopic dosage form and the study on its potential for stunting conditions are highly needed. The concentrate was encapsulated into granules by using different fillers with Florite as desiccant. Physicochemical evaluations were conducted using Differential Scanning Calorimetry (DSC), X-ray Diffraction (XRD), and Scanning Electron Microscopy (SEM). An in vivo anti-stunting study was performed using zebrafish as an animal model. The results indicated that encapsulation using Avicel PH 102 as filler, either with or without addition of Florite 10%, provided good stability after 5 months of storage. Antistunting activity test showed that, after induction by rotenone, treatments with deep seawater either in form of concentrate water or granule improved the stunted condition of the zebra fish embryos shown as the improvement in the hatching rate, brain length, and body length. Deep seawater concentrate was successfully formulated into granules which provided moisture protection of its highly hygroscopic nature as well as antistunting activity.

## Introduction

Vitamins and minerals play an important role in human growth and development as well as in maintaining optimal health. Micronutrient deficiencies are often caused by inadequate intake and an insufficient supply of vitamins and minerals. If micronutrient deficiency occurs in children, it can cause stunting^1,2^, in which children are shorter than the average for their age. Additionally, stunting is not only a matter of height but also about children who are not developing well, physically or mentally. The impact of stunting can also affect neurocognition, namely stunted brain, thus hindering development, as well as being a symptom of past poverty and a predictor of future poverty^3,4^. The direct and long-term impacts of stunting on children include increased morbidity and mortality rates, lower development and learning capacity, and increased risk of infectious diseases in adulthood. In the end, it can reduce productivity and global economic capacity^5^. Stunting intervention strategies are generally carried out on pregnant women and toddlers by providing supplements or micronutrients, vitamins, and adding protein in daily consumption^1,2^.

Deep seawater concentrate has enormous potential to be used as raw material of supplements as a source of minerals in multivitamins^6^. As the demand for minerals increases, synthetic mineral sources from mining are diminishing, making concentrated seawater a viable alternative. Deep seawater is a natural resource that is abundantly available and will never run out, without the need of cultivation. It produces a product known as bittern – a concentrated seawater solution with high mineral content of potassium (K), calcium (Ca), and magnesium (Mg). Currently, seawater concentrate has been utilized in the health sector as a raw material for supplements and multivitamins^6^.

Indonesia has a very long coastline to which 43052.10 hectares of salt land, with approximately 59.7% dedicated to salt production^7^. Despite the abundant seawater sources, the potential of these materials has not been extensively studied. In the pharmaceutical sector, it is imperative to characterize and standardize raw materials thoroughly to guarantee the safety and quality of the final product. The quality can be assessed through the physical, chemical, and biological parameters^8,9^. Preliminary study on the quality of deep seawater to enhance the insights concerning the safety and quality of deep seawater concentrate for fabrication has been conducted and validated by using the standards which adopted from those available for drinking water. Quantitative determination of the mineral/metal content can be performed using ICP-OES (Inductively Coupled Plasma – Optical Emission Spectrometry) and X-ray fluorescence (XRF)^10,11^.

The concentrated deep seawater to be marketed as multivitamins faces the challenge due to its bulky volume. As a solid preparation, stability of pharmaceuticals and nutraceuticals with highly content of minerals which are hygroscopic in nature is greatly affect their shelf life and therapeutic performance^12–14^. The hygroscopic character of pharmaceutical raw materials also significantly affects their stability, appearance, and efficiency. With regard to cover the stability problem of hygroscopic material, Mabrouki (2022) has conducted research related to the formulation and development of tablets from *Herniaria Glabra* L extract, which is known to be hygroscopic, and improved its properties and physical stability by using a film coating system^15^. Other study revealed that granulation provides good homogeneity as well as stability of product thus more practical and easy in storage and distribution^16^. Study on the effect of high mineral content in solid material proved that highly hygroscopic properties of the powder are due to susceptibility in absorbing moisture^17^. Zhang et al. have conducted research related to the hygroscopic properties of the mineral content by studying the properties of sodium and potassium salts during storage^18^. Therefore, it is important to study and classify the problem of materials with highly hygroscopic properties and provide information on the handling of these materials^19^.

Thus, this study aimed to provide information on fabrication of high-quality granules of deep seawater mineral concentrates which bring high hygroscopic property by nature as multivitamins supplementation in stunting management. The information provided includes formulation technology aspects to improve the stability of granules, as well as study on antistunting activity against stunted zebrafish as animal model. The use of zebrafish as an animal model has demonstrated high success in drug discovery research, preclinical drug development, pathophysiology, and pharmacology studies of human diseases^20,21^. The advantages of this method are rapid development embryos, easy manipulation of the genome, laying eggs that provide hundreds of embryos, low maintenance, and owing 70% homology with human genes^22,23^. Previous research related to stunting activity using zebrafish (Primihastuti et al.) revealed that a reduction in body length of the stunted zebrafish can be easily observed during treatment^24,25^. Deep seawater concentrate encapsulation technology increases product stability, which leads to an increase in micronutrient availability in the form of multivitamins to provide the nutrients needed to support optimal growth and development in stunted children^26^.

## Results

### Chemical analysis of deep seawater concentrate

Analyses were conducted to qualitatively and quantitatively determine the chemical content of the samples. This was performed using both X-Ray Fluorescence (XRF) and Inductively Coupled Plasma–Optical Emission Spectrometry (ICP-OES), as shown in Table 1.

**Table 1:** Chemical content analysis of deep seawater concentrate using XRF and ICP-OES.

Essential minerals				
Elements	Samples (mg/l)		Market product (mg/l)	
	XRF	ICP-OES	XRF	ICP-OES
Mg	138,000	61817.1466	53,200	1656.2590
Cl	626,000	*	160,000	*
K	82,300	72888.7852	21,500	172141.0630
Ca	8370	527.9098	0	94.4760
Na	0	350.7511	0	643.0545
B	0	110.1492	0	154.7290
Cr	0	< 0.0001	2.56	< 0.0001
Cu	18.0	< 0.0001	7.46	< 0.0001
Fe	26.1	< 0.0001	0	0.0095
Mn	0	< 0.0001	0	4.3815
Zn	0	< 0.0001	0	1.0785
Toxic minerals				
Al	0	< 0.0001	0	6.4350
Tl	0	< 0.0001	0	< 0.0001
As	0	< 0.0001	0	< 0.0001
Hg	0	< 0.0001	0	< 0.0001
Cd	0	< 0.0001	0	< 0.0001
Pb	0	< 0.0001	0	< 0.0001
Non-essential and non-toxic minerals				
S	79,000	*	20,700	*
Br	19,100	*	3010	*
Si	5340	*	1870	*
Rb	790	*	49.9	*
Sn	325	< 0.0001	78.6	< 0.0001
Ta	31.2	*	11.4	*
In	26	< 0.0001	0	< 0.0001

*Not conducted.

### Fabrication and stability study of deep seawater granules

The fabrication of deep-seawater concentrate granules was conducted using two types of fillers to which l actose anhydrous and Avicel PH 102, to evaluate the effects of different types of fillers. Based on the organoleptic assessment, it was observed that the granules were white for G-Av and G-Av + Fl, whereas G-Lact and G-Lact + Fl were white-yellowish. They were odorless and showe granular form in morphology.

- G-AV : Avicel Granules.
- G-AV + FL : Avicel Granules + Florite 10%.
- G-Lact : Lactose anh. Granules.
- G-Lact + Fl : Lactose anh. Granules + Florite 10%.

Granules G-Av, G-Av + Fl, and G-Lact + Fl exhibited good stability during storage at room temperature, whereas G-Lact granules were unstable. The formula G-Lact, which uses lactose anh. as a filler without the addition of a drying agent, begins to liquify after 3 days of storage.

**Fig. 1:**
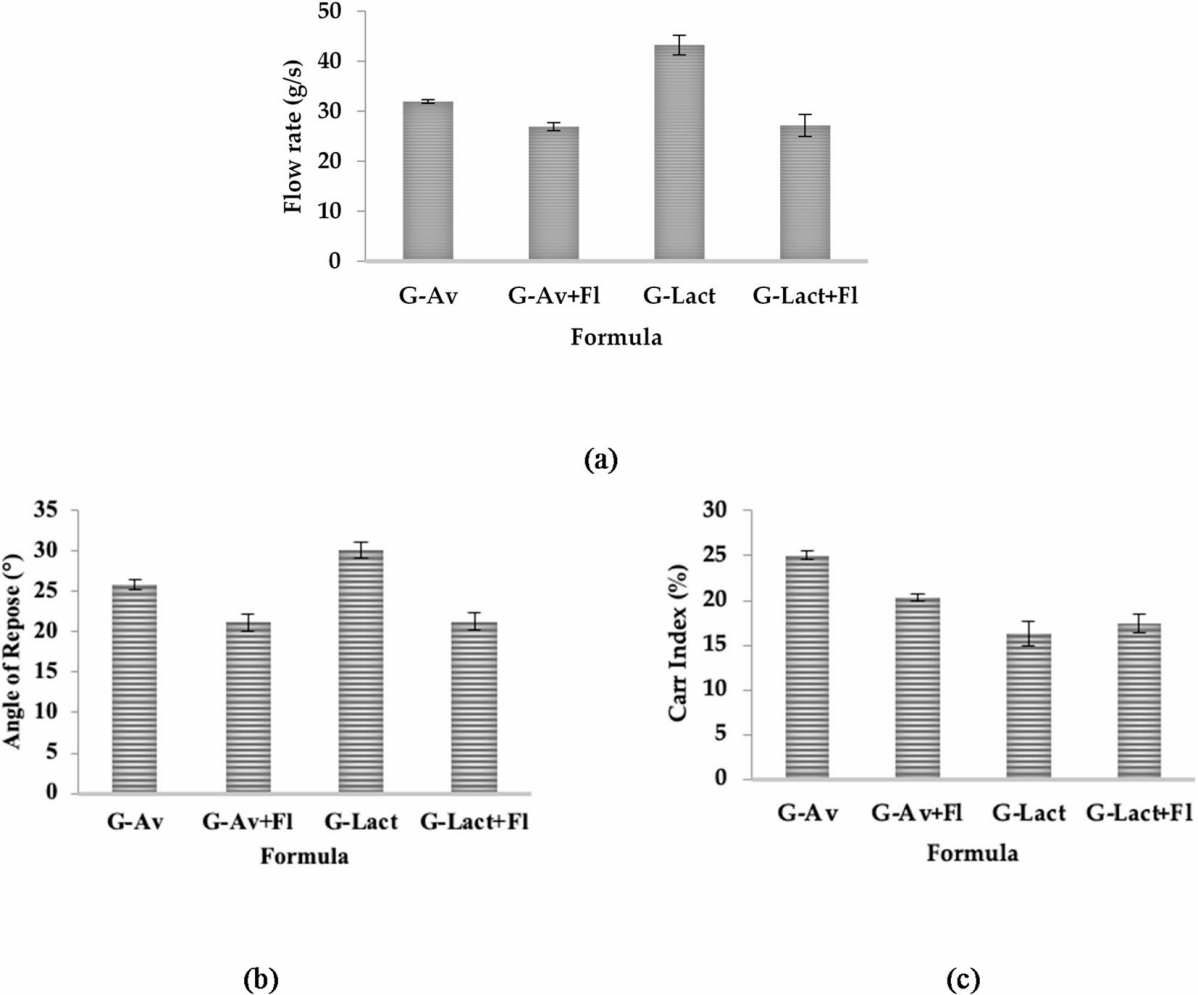
Effect of Florite addition as drying agent on (**a**) Flow Rate, (**b**) Angle of Repose, and (**c**) Carr’s Index Value of granules.

Evaluation of the flow rate of the granules showed that the addition of Florite as a drying agent improved the flowability by using either Avicel or lactose anh. as a filler (Fig. 1a). This finding is in line with the results for the angle of repose, as shown in Fig. 1b. The compressibility/Carr index results are shown in Fig. 1c.

**Fig. 2:**
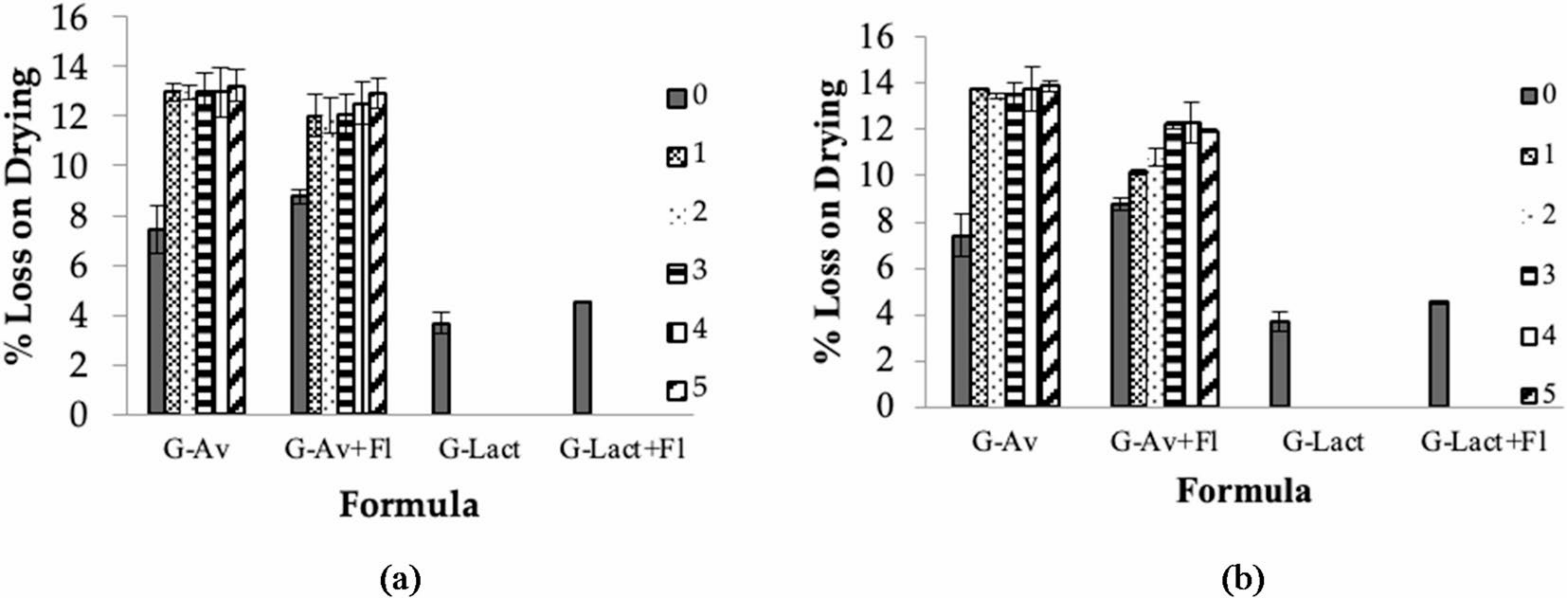
Effect of Florite addition as drying agent on LoD values of granules (**a**) room temperature and (**b**) climatic chamber after stability study 0, 1, 2, 3, 4, and 5 months of storage.

Following the preparation of the seawater concentrate granules, their physical stability was analyzed using the LoD parameter based on selectivity for moisture protection. The results in Fig. 2 showed the moisture stability of the granules under two different conditions after 5 months of storage.

### Physical stability study of granules using instruments

The dried mineral granules from the seawater concentrate, which were successfully produced using the optimization formula in the previous stage, were introduced into a stability study for 5 months of storage, and the stability parameters of the granules were analyzed using instruments. Hygroscopicity, as the main problem of solid materials with high mineral content, was analyzed in terms of thermal behavior by DSC, crystallinity by XRD, and confirmation of morphological performance by SEM.

#### Thermal analysis by DSC

**Fig. 3:**
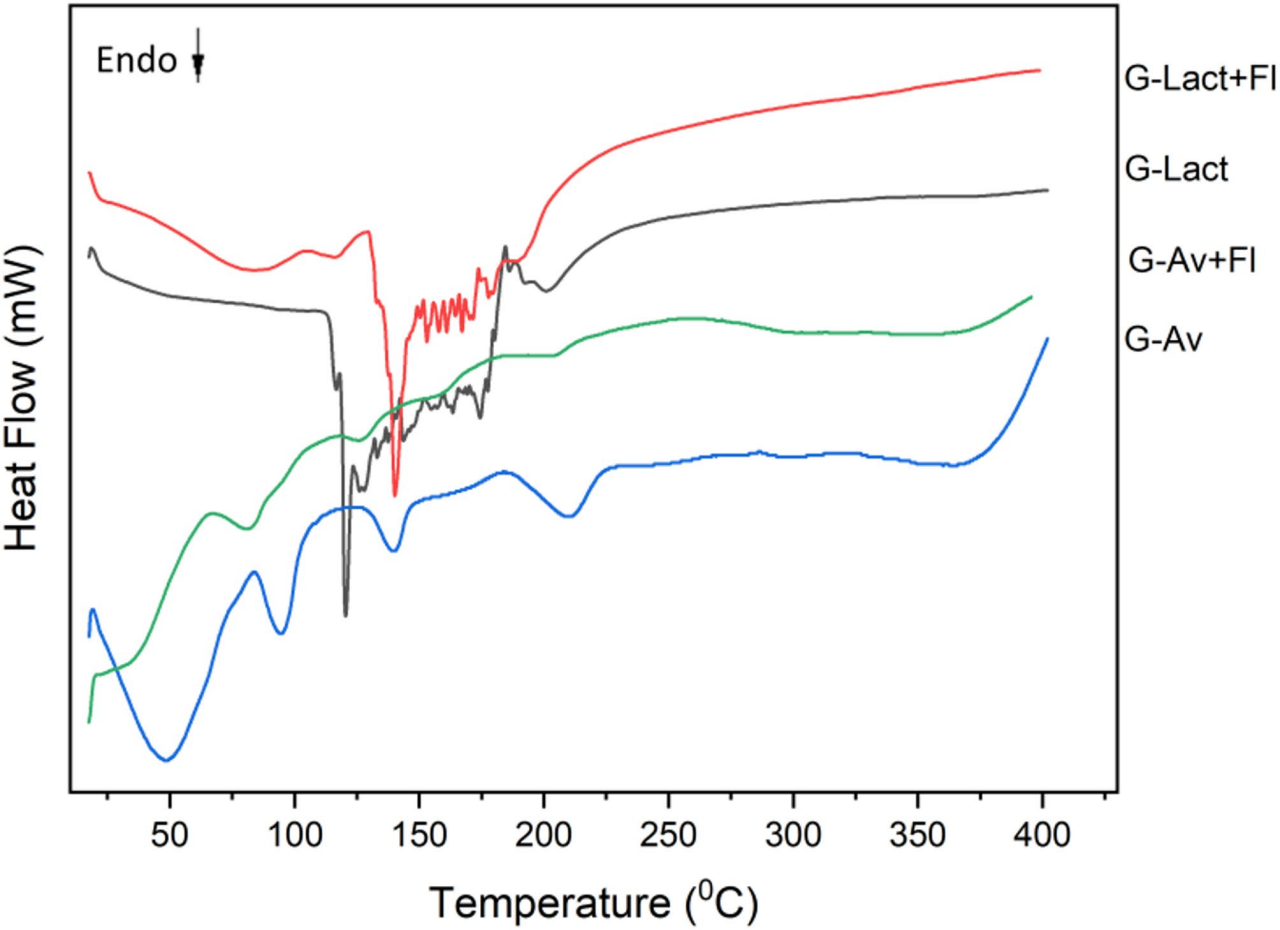
DSC thermogram of deep seawater granules using different filler.

The Thermal behavior analysis were conducted on all seawater granule formulations, including lactose anh. granules (G-Lact), lactose anh. granules withFlorite 10% (G-Lact + Fl), Avicel granules (G-Av), and Avicel granules withFlorite 10% (G-Av + Fl) (Fig. 3).

**Fig. 4:**
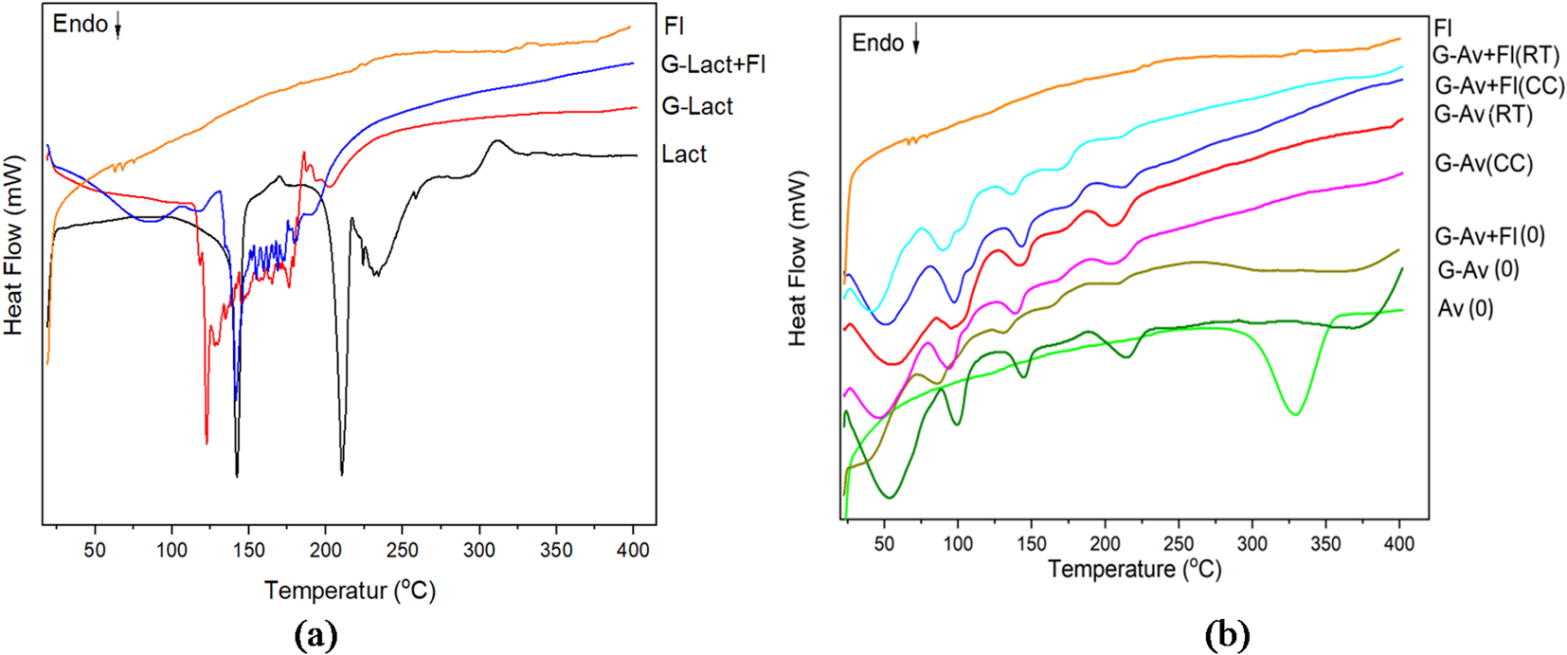
DSC thermogram of deep seawater Granules using (**a**) Lactose anh. and (**b**) Avicel PH 102 as fillers at day of formulation (0) and after five months of storage at room temperature (RT) and Climatic Chamber (CC).

Stability studies by the thermal behavior observation to the granules were conducted after 5 months of storage (Fig. 4). Studies on granules using lactose anh. as filler were performed with formula lactose anh. granules (G-Lact), lactose anh. granules with Florite 10% (G-Lact + Fl), as well as lactose anh. and Florite powder for comparison (Fig. 4a). Accordingly, stability studies on granules with Avicel PH 102 as filler were conducted with formula Avicel granules (G-Av) and Avicel granules with Florite 10% (G-Av + Fl) after formulation (0) and after 5 months of storage under two conditions: room temperature (RT) and climatic chamber (CC), as well as to Avicel PH 102 (Av) powder for comparison (Fig. 4b). During the stability study, it was revealed that the granules with lactose anh. as a filler melted at approximately 2 weeks of storage, and thus could not undergo further stability studies.

#### XRD analysis

**Fig. 5:**
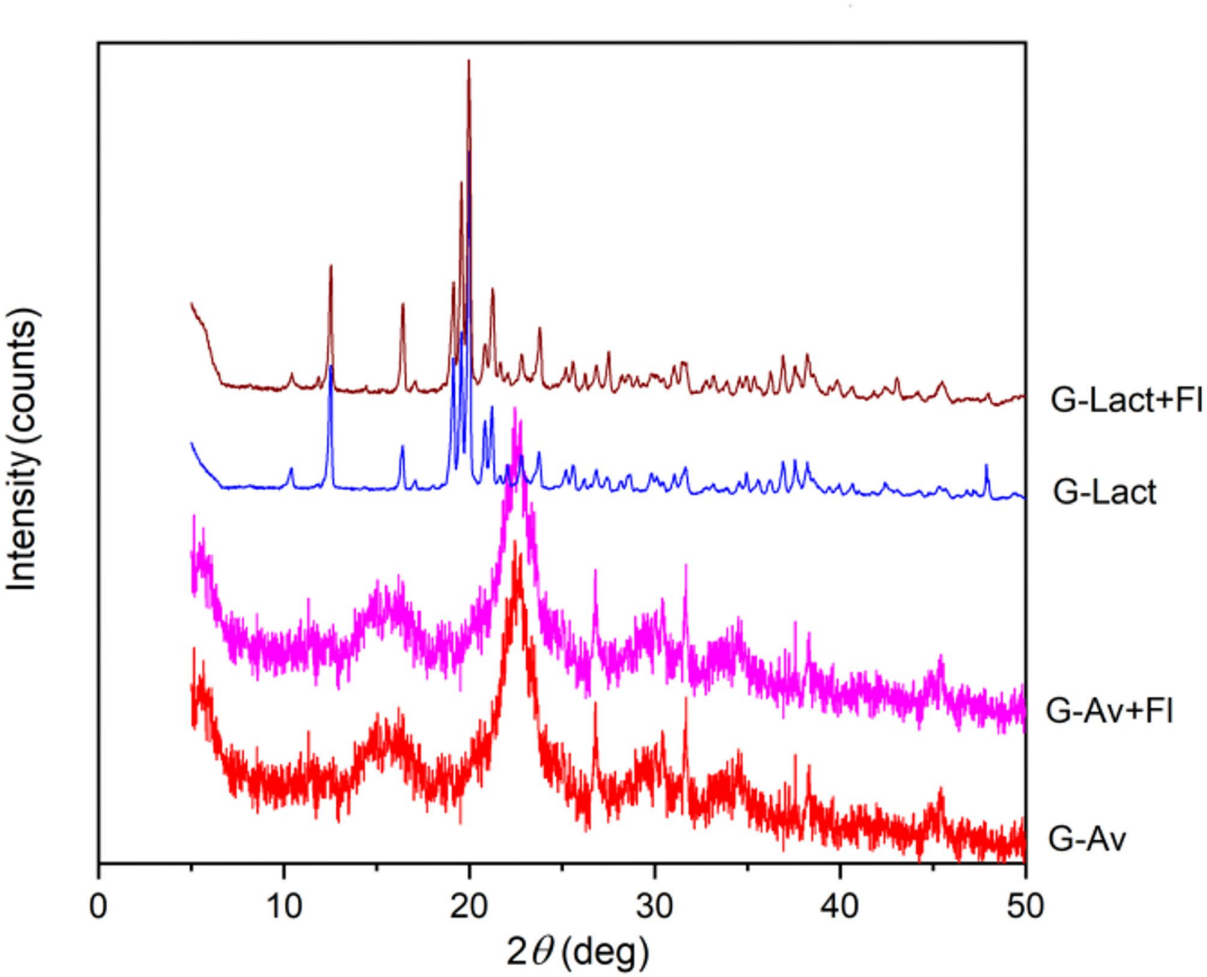
XRD diffraction patterns of Deep Seawater Granules using different fillers.

The XRD diffraction patterns of deep seawater granules using fillers of Avicel, Avicel with Florite 10%, lactose anh., and lactose anh. with Florite 10%, are shown in Fig. 5.

**Fig. 6:**
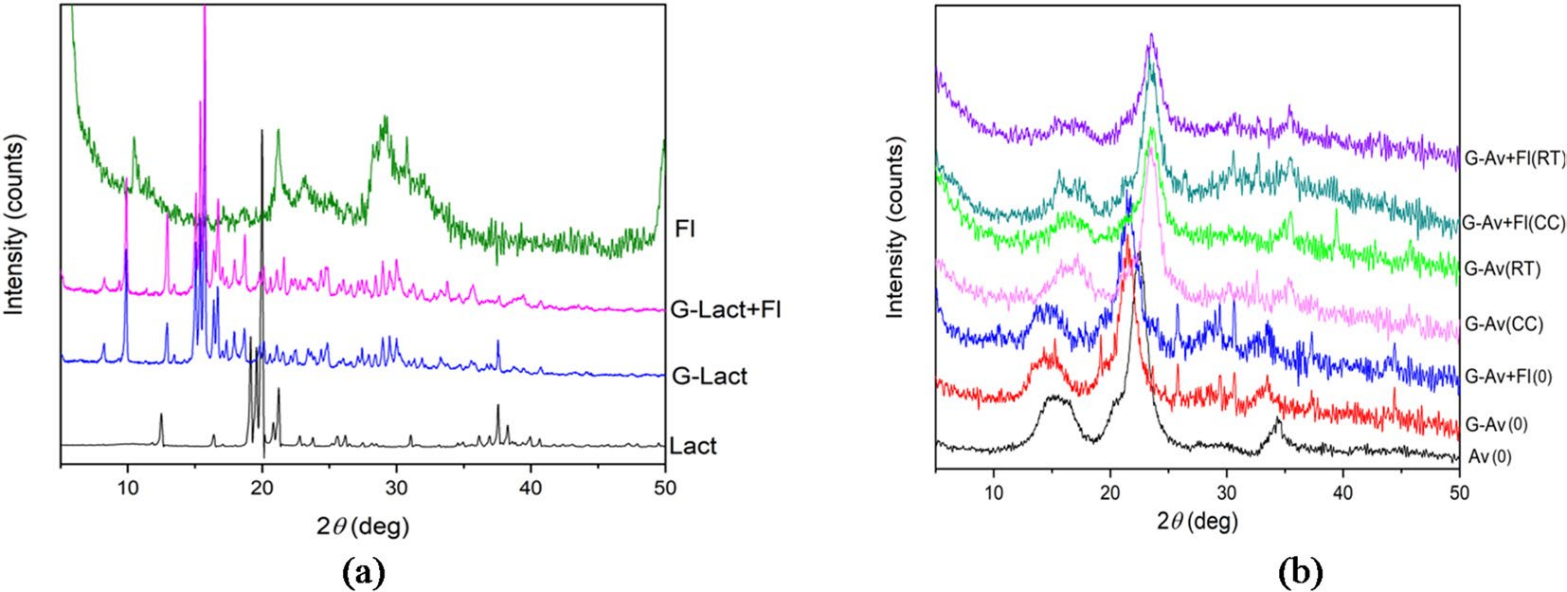
Difractograms of deep seawater Granules using (**a**) Lactose anh. and (**b**) Avicel PH 102 as fillers at day of formulation (0) and after five months of storage at room temperature (RT) and Climatic Chamber (CC).

The results of the stability study of the XRD pattern were conducted using Avicel as a filler because of the failure of the lactose anh. granule formulation to stand during the study (Fig. 6a). Characterization by XRD during stability studies on granules with Avicel PH 102 as a filler was conducted on Avicel granules (G-Av (0)) and Avicel granules with Florite 10% (G-Av + Fl (0)) after formulation and after 5 months of storage under two conditions: room temperature (RT) and climatic chamber (CC) as well as to Avicel PH 102 (Av) powder for comparison (Fig. 6b).

#### Morphology study by scanning electron microscope

**Fig. 7:**
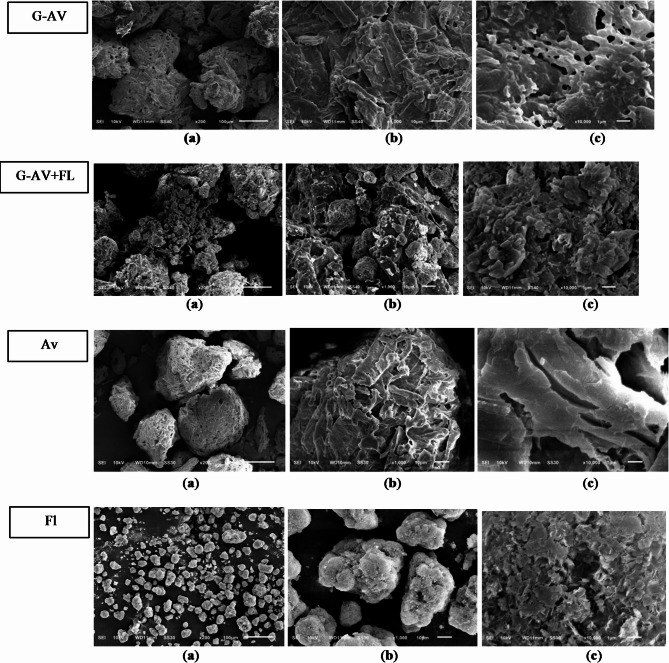
Morphology of deep seawater granules using Avicel as filler (G-Av); Avicel PH 102 with Florite 10% (G-Av + Fl); Avicel powder (Av) andFlorite powder (Fl) using magnification of (**a**) 200 x; (**b**) 1000 x; and (**c**) 10,000 x.

**Fig. 8:**
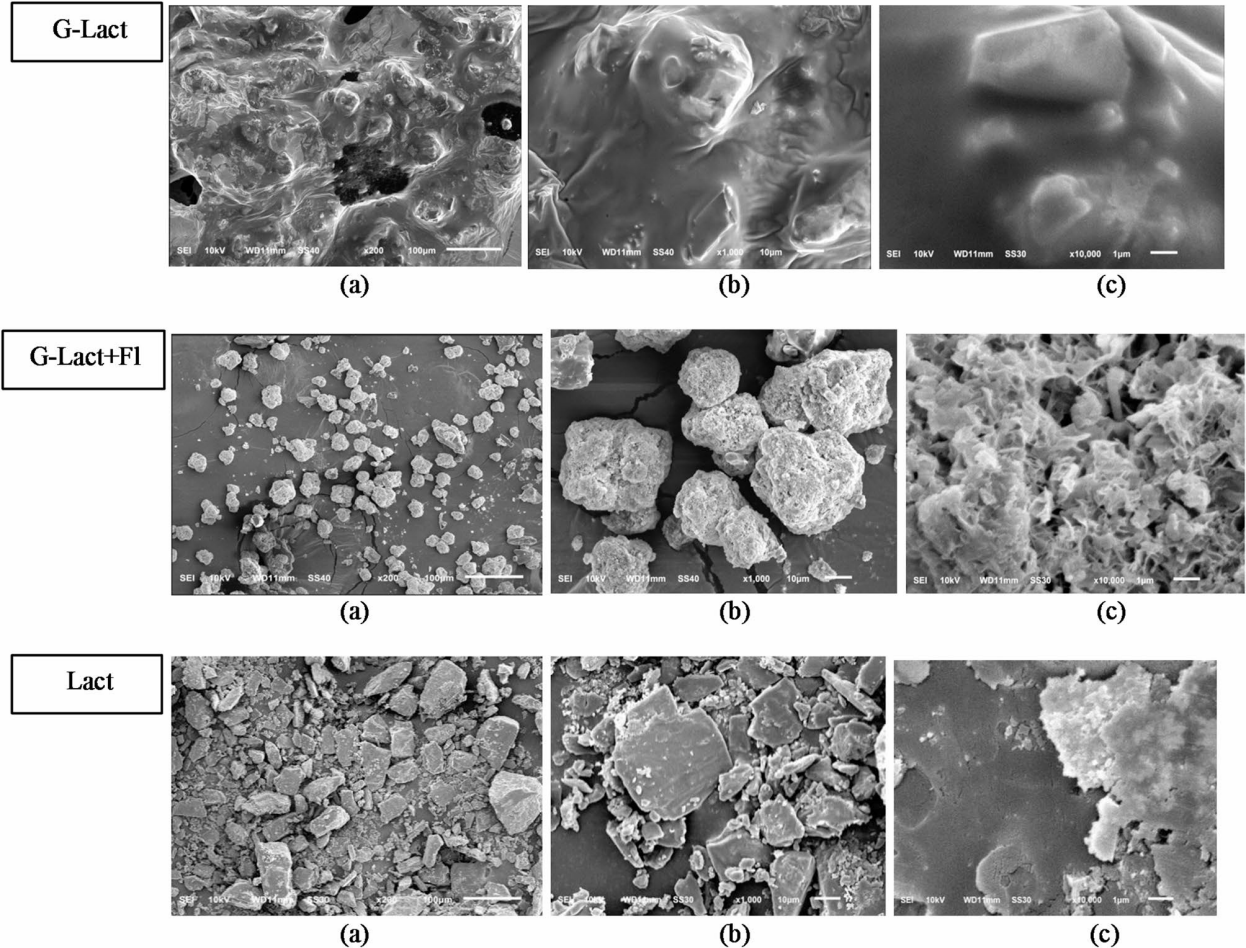
Morphology of deep seawater granules using lactose anh. as filler (G-Lact); lactose anh. with Florite 10% (G-Lact + Fl); and lactose anh. (Lact) powder using magnification of (**a**) 200 x; (**b**) 1000 x; and (**c**) 10,000 x.

**Fig. 9:**
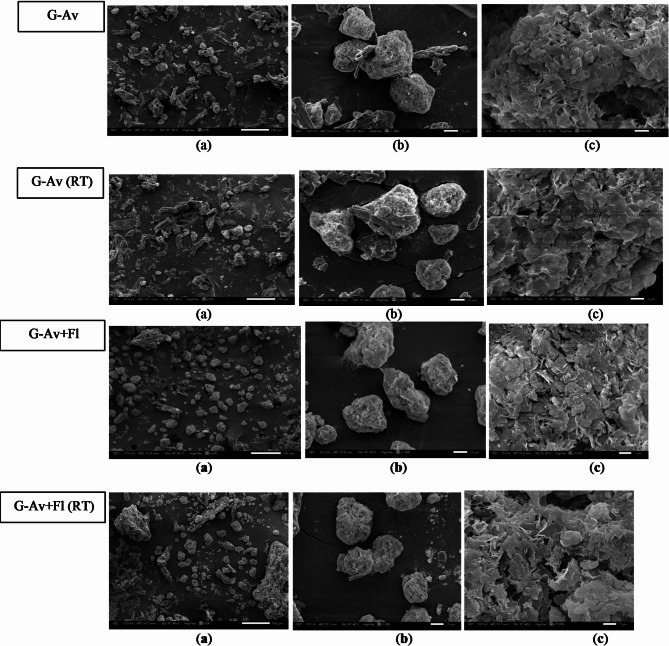
Morphology of deep seawater granules after 5 months of stability study from granule using Avicel PH 102 as filler (G-Av); Avicel PH 102 with Florite 10% (G-Av + Fl); at room temperature (RT) and at climatic chamber (CC) using magnification of (**a**) 200 x; (**b**) 1000 x; and (**c**) 10,000.

Observations of the morphology and particle surface of deep seawater granules and standards conducted using Scanning Electron Microscopy (SEM) are shown in Figs. 7, 8 and 9.

### Antistunting activity study

The antistunting study was conducted in vivo using zebrafish (*Danio rerio*) embryos because of fast embryo development with the ability to provide hundreds of embryos from one individual egg. Not to mention the high homology (70%) with human genes^22,23^. In studying anti-stunting activity, parameters to be observed in this experiment were hatching rate, brain length, and body length.

#### Hatching rate

**Fig. 10:**
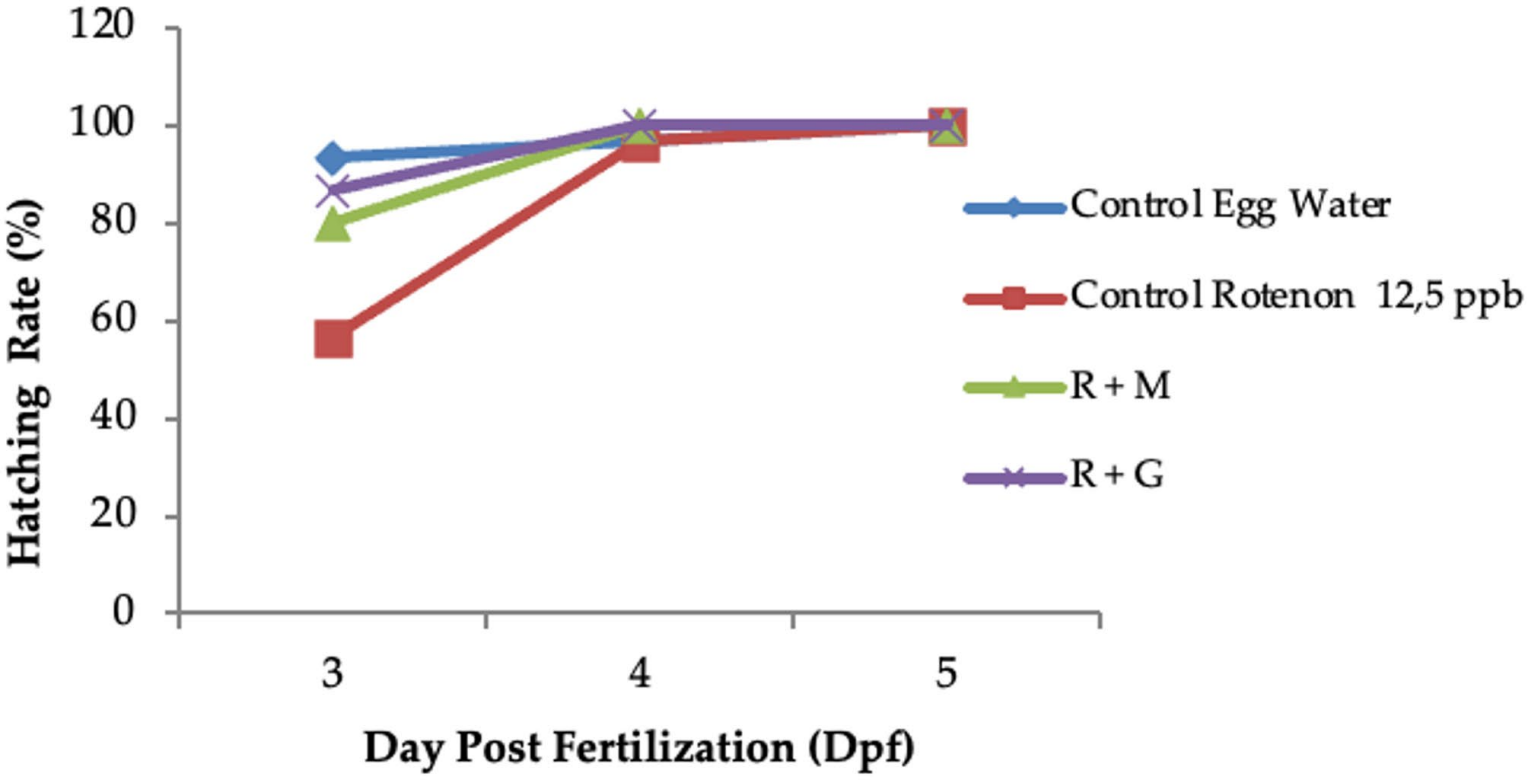
Percentage of hatched zebrafish eggs (*n* = 30) after treatment by rotenone continued with concentrate mineral (R + M) compared those induced by rotenone continued with Granule (R + G) compared with controlled Egg in water and controlled egg treated with rotenone (12.5 ppb) as Stunting Inducer agent.

The hatching rate of the zebrafish embryos was observed at 3–5 dpf (days post-fertilization) to evaluate the possibility of delayed egg hatching due to interference with enzyme activity and embryo movement, as shown in Fig. 10.

#### Brain length

**Fig. 11:**
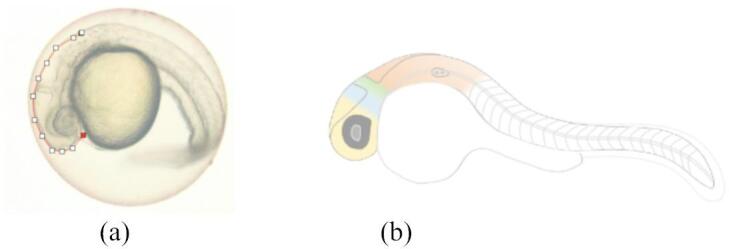
Visual Zebrafish’s Brain Length measurement (**a**) Macroscopic observation of zebrafish’s brain after treatment (white dots with red line); (**b**) Zebrafish’s brain division based on reference^27^.

**Fig. 12:**
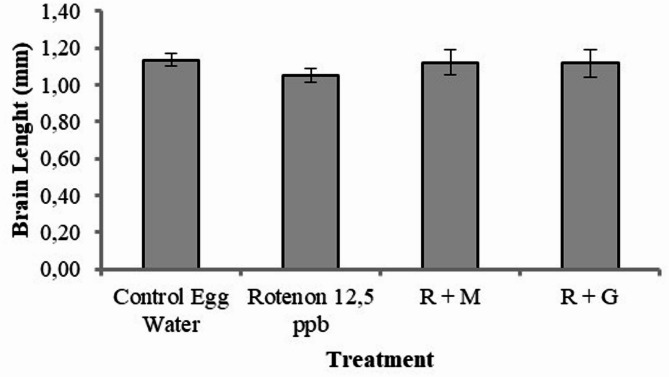
Brain length of Zebrafish embryos at age of 24 hpf (data are mean ± SD, *n* = 30).

The effect of sample treatments on the brain length of the embryo is one of main parameters in this antistunting study due to the stunting effect on neurocognitive responses such as dwarf brain and hindered growth development^3^. Brain lengths were measured at 24 hpf (hours post-fertilization), to which the division of the brain is still easy to be visually observed, as shown in Fig. 11. The brain length of the embryo from the zebrafish group treated with rotenone (as a stunting inducer agent) was the shortest compared to the group treated with rotenone continued with seawater concentrate (R + M). Surprisingly, the group treated with rotenone and continued with deep seawater granules (R + G) had a similar length to that of the control group (Fig. 12).

### Body length

**Fig. 13:**
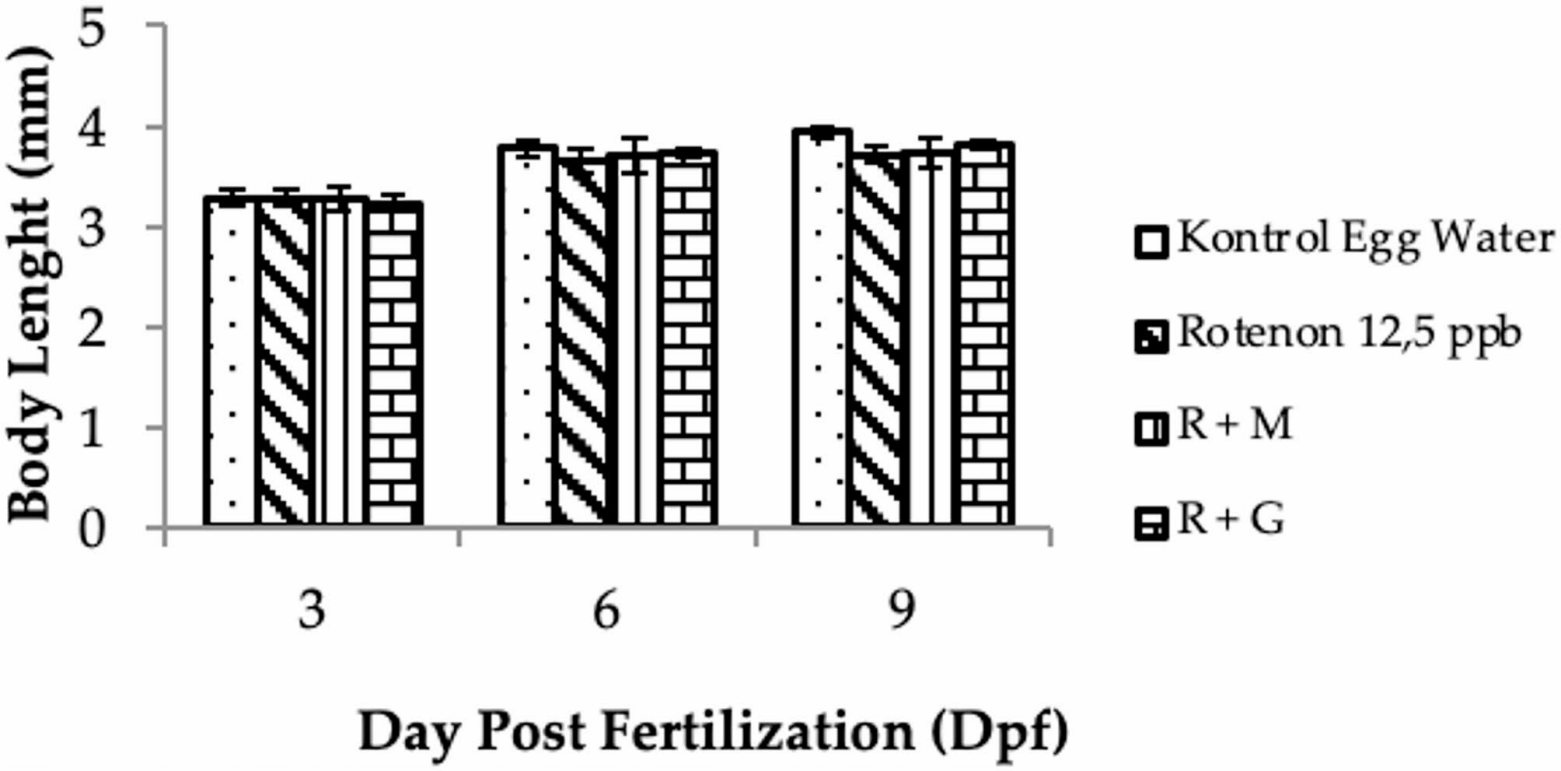
Body length of Zebrafish after treatments (data are mean ± SD, *n* = 30).

**Fig. 14:**
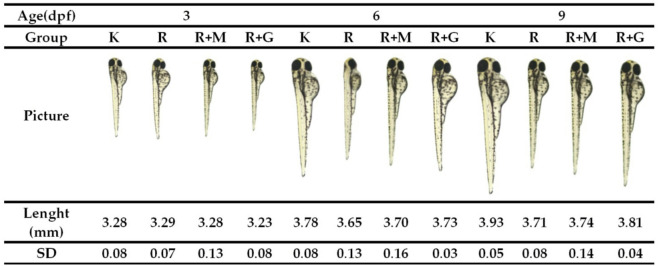
Picture body length of Zebrafish after treatments of : controlled Egg in water (K); controlled egg treated with rotenone (12.5 ppb) as Stunting Inducer agent (R) ; rotenone continued with deep seawater concentrate (R + M); rotenone continued with Granule of deep seawater concentrate (R + G).

The effect of treatments with rotenone and deep seawater concentrate on body length of the animal model is the important parameter to be studied since, based on WHO, stunting is defined as a decrease in body length compared with normal standard in child’s growth^24^. Body length measurement was conducted at 3, 6, and 9 dpf, which was based on the analogy that 6 dpf is equal to a child aged 6 years old and 9 dpf is equal to the child at 8 years^25^. The results of measurement on a zebrafish’s body length showed that the treatments had affected the body length (Figs. 13 and 14).

### Cranial cartilage development

**Fig. 15:**
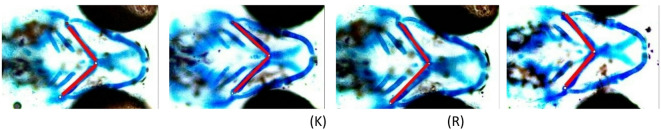
CH -Angle of 9 Dpf Zebra fish embryos at 100x magnification after different treatments ; controlled Egg in water (K); controlled egg treated with rotenone (12.5 ppb) as Stunting Inducer agent (R); rotenone continued with Granule of deep seawater concentrate (R + G); rotenone continued with deep seawater concentrate (R + M) (data are mean ± SD, *n* = 30). (K), (R), (R + G), (R + M).

Parameter of Cranial Cartilage morphology development is important in stunting case since it directly connected to bone development and growth^3^. Stunting condition in this experiment was confirmed by the decrease in bone growth from zebra fish group treated by rotenone (R) as stunting inducer. Ceratohyal structure (CH) of different treatment on Zebra fish embryo after 9 Dpf (Fig. 15).

**Fig. 16:**
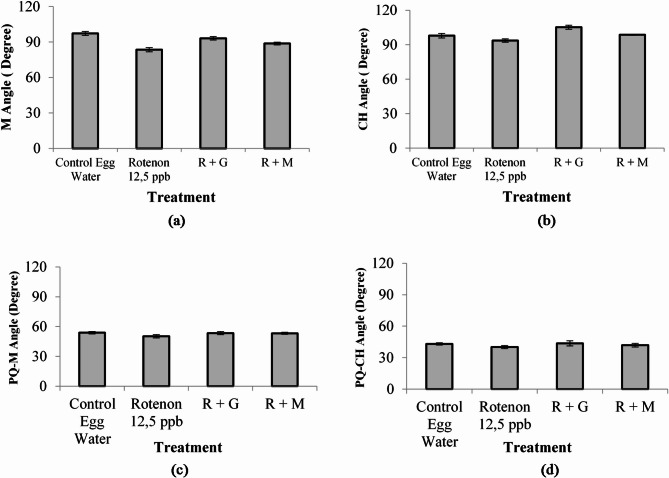
Cranial Cartilage measurement of the embryos after different treatments from different angle views; M-Angle (**a**); CH Angle (**b**); PQ-M Angle (**c**); and PQ-CH Angle (**d**) (data are mean ± SD, *n* = 30).

Embryonic Cartilage development of zebra fish are evaluated not only by ceratohyal (CH) parameter but also from Meckel angle view (M), palatoquadrate and Meckel cartilage angle view (PQ-M), as well as palatoquadrate and ceratohyal view (PQ-CH) (Fig. 16). The result showed that treatment by deep seawater granule revelaed a significant difference (*p* < 0,05) compared with that of controle egg water as well as negative control (treated by rotenone 12.5 ppb) when observed as parameters of ceratohyal (CH), Meckel angle view (M), palatoquadrate and Meckel cartilage angle view (PQ-M), as well as palatoquadrate and ceratohyal view (PQ-CH).

## Discussion

### Quantitative chemical analysis of deep seawater concentrate

The analysis of deep seawater concentrates were conducted by using X-Ray Fluorescence (XRF) and Inductively Coupled Plasma–Optical Emission Spectrometry (ICP-OES) methods, owing to their capability of simultaneous multi-element detection, detection speed, high sensitivity, and wide detection range^28–30^. The results from these two instruments were compared to study their ease and sensitivity for use in mineral content analysis. The results indicated that the seawater concentrate sample or marketed products contained the major components of magnesium (Mg), chloride (Cl), potassium (K), calcium (Ca), sodium (Na), and boron (B) (Table 1).

The difference between the mineral content detected from marketed products compared with deep seawater sample when analyzed using XRF and ICP-OES, either in qualitative or quantitative quantities of the elements, contributed to the variation in sample treatment between XRF and ICP-OES analysis. In the XRF method, the samples do not need to undergo destruction due to their capability to analyze both solid and liquid samples without prior destruction or dissolution. However, this can result in a higher matrix effect, which potentially affects the results^28^. Seawater concentrate, which was formerly processed into a concentrated form, can precipitate salts during storage^31^. ICP-OES analysis cannot be conducted directly into solid samples. The sample must be dissolved, requiring the digestion of solids with acid^10^. Mostly, aqua regia, consisting of HNO_3_ and HCl in a 1:3 ratio, must be dissolved, which requires the digestion of solids with acid. Acid digestion can alter the form of elements, some of which are not easily soluble or ionized. It can break down complex compounds or minerals, releasing elements^10^. The differences in sample treatment and matrix composition of the sample of XRF and ICP-OES analyses can affect the results, potentially leading to discrepancies between the outcomes. XRF can detect a greater number of elements than ICP-OES can. However, in terms of trace elements, ICP-OES demonstrates the ability to detect elements at lower concentrations, ranging from 0.0365 to 18,625.8 mg/L, whereas XRF operates within a higher detection range, from 6.61 to 188,319 mg/L. In contrast, XRF is more suitable for detecting a broader range of elements with higher concentrations.

Based on these data, the seawater concentrate sample revealed to contain minerals that are essential for supplementation, including major content of magnesium (Mg), chloride (Cl), potassium (K), calcium (Ca), iron (Fe), copper (Cu), and sodium (Na), which meet the requirements for daily mineral intake as multivitamin supplements. Furthermore, heavy metals such as mercury (Hg), lead (Pb), cadmium (Cd), and arsenic (As) were not detected in the sample, indicating their safety as raw materials for multivitamin source.

### Stability study of deep seawater granule formulations

Seawater concentrate is a concentrated liquid of deep seawater rich in minerals that is required for supplement/multivitamin. In terms of stability, raw materials of pharmaceutical dosage forms will be better in solid form either as powders or granules because they are not only stable, but also provide other advantages, such as ease of practical use, handling, and distribution. Deep seawater, as a raw material for multivitamins, most of which are minerals, changes from liquid to solid in the form of granules. Drying and stabilizing granules during storage has become a major challenge. Previous research has shown that a high mineral content makes solid preparations hygroscopic. To overcome this problem, it is necessary to select an optimum diluent and desiccant/drying agent. Lactose anh. has been used as a filler for the reason of affordable price to reduce production unit costs while microcrystalline cellulose was expected to provide better drying capability. The study of multifactor influencing granule stability has been elaborated to provide comprehensive data on the development of granules of deep seawater as a raw material for multivitamins.

Upon formulation, the evaluation of the flow rate of all granules revealed good flow properties (exceeding 10 g/s flow rate, Fig. 1a). Among other formulations, lactose anh. granules (G-Lact) showed the highest flowability owing to their larger particle size compared to that of microcrystalline cellulose (Avicel PH 102). This result is in line with a previous finding that large particles exhibit better flowability^32^.

In contrast, the angle of repose determination exhibited better flowability for Avicel granules than for those using lactose anh. as a filler (Fig. 1b). The high values of the angle of repose from lactose anh. granules were attributed to the hygroscopic nature of the material, which caused the granules to absorb moisture from the air and stick together in the funnel^33,34^. The data from the Carr’s Index and flow rate test align with theoretical expectations, indicating good flow properties for G-Lact. However, the results from the angle of repose test showed an anomaly, with the highest angle indicating poor flow. This discrepancy can be attributed to the hygroscopic nature of G-Lact, which significantly influences its flow behavior. The hygroscopicity causes the G-Lact particles to attract moisture, leading them to adhere to each other. As a the result, when measuring the angle of repose, the particles do not disperse evenly, but rather clump together, creating a peak that results in a higher angle of repose. This interaction between particles due to their hygroscopicity explains the observed anomaly in the angle of repose test^33^.

The flowability increased as measured by smaller values of angles to less than 25 after the addition of Florite as a dying agent. These findings support those of a previous study, which found that the moisture content coated the particles to enhance their sticking ability, thus increasing the particle interaction and increasing the angle of repose.

Based on the compressibility/Carr’s index results shown in Fig. 1c, all formulations exhibited good compressibility values ranging from 5 to 20%, except for G-Av (25.71%). Compressibility refers to the ability of a material to reduce its volume under a certain pressure^35^. Formula G-Av had smaller granule sizes than those of the other formulations. Granule size affects compressibility: smaller granules tend to have higher compressibility percentages, whereas larger granules exhibit lower percentages^36^.

The production of granules requires consideration of stability and ease of distribution. To enhance stability, a desiccant is essential to counteract the hygroscopic nature of the mineral content in the granules. In this experiment, Florite was used as the desiccant at a concentration of 10%. It is a synthetic calcium silicate with an outstanding liquid absorption capability up to approximately five times its weight. It has also been reported to exhibit improved compressibility properties^37^.

The G-Av, G-Av + Fl, and G-Lact + Fl granules remained stable at room temperature, whereas the G-Lact formula melted on the third day of storage. Granules using Avicel as a filler (G-Av and G-Av + Fl) demonstrated good stability for 5 months of storage at room temperature (Fig. 2a) or during stress in a climatic chamber (Fig. 2b). In addition to serving as a filler, Avicel also provides adsorbent capability, thus reducing the hygroscopic properties^38,39^.

Accordingly, the granule formula using lactose anh. as fillers G-Lact and G-Lact + Fl (lactose anh. filler and Florite as a desiccant) melted in the first month and could not continue the stability testing (Fig. 2a and b). At the early stage of process evaluation, G-Lact granules were obtained and were more hygroscopic than G-Lact + Fl granules, indicating thatFlorite affected the hygroscopic properties of the resulting granules. Nevertheless, these granules could not survive and liquified after 2 weeks of storage. It can be concluded that the filler type has a more dominant influence on the formulation than the desiccant.

The results indicated that Avicel, a model microcrystalline cellulose derivate, can effectively maintain the stability and quality of microscopic granules with a high mineral content in seawater concentrate. Thus, moisture protection of deep-sea water in the form of a solid material (granule) has been successfully developed.

### Thermal behavior analysis of granules by DSC testing

Differential scanning calorimetry (DSC) was used to observe the melting point shifts and the transition points of the samples. DSC can also be used as a parameter for stability testing^40^. The thermal analysis of deep seawater granule formulations – including Avicel granules (G-Av), Avicel granules withFlorite 10% (G-Av + Fl), lactose anh. granules (G-Lact), and lactose anh. granules withFlorite 10% (G-Lact + Fl) – were conducted by using DSC (Fig. 3). The results in Fig. 3 show that the addition of a drying agent (Florite) improves the melting point of the granules to become higher. Notably, there were differences in the melting peaks between the granules with lactose anh. and those with Avicel as fillers. This shift in melting point correlated with enhanced stability of the granules containingFlorite 10%, showing greater thermal stability. Similarly, differences in thermal transition points were observed between lactose anh. granules (G-Lact) and lactose anh. granules withFlorite (G-Lact + Fl), indicating that the addition ofFlorite positively affected the stability of the granules. The melting point ofFlorite is 1.540 °C, that of lactose anh. is 201–202 °C ^,^ and that of Avicel is 260–270 °C^41,42^.

Distinct differences were observed in the thermal behavior of the two formulations (Fig. 4a and b), which were due to the different physical properties of lactose anh. (crystalline) and Avicel/microcrystalline cellulose (semicrystalline). This hypothesis is discussed in the section on XRD analysis. During the stability study, Avicel granules (G-Av) and Avicel granules withFlorite (G-Av + Fl) showed a more robust formulation, suggesting that the type of filler significantly influenced granule stability. Granules with lactose anh. as a filler could not withstand the stability testing and began to melt in the second week of storage; thus, further observation on stability testing could not be continued (Fig. 4a). The hygroscopic properties of lactose anh. granules are not only due to the chemical properties of lactose anh. but also due to the larger particle size and porosity of the granules. The granules using Avicel PH 102 as a filler successfully revealed good stability until 5 months of storage, showing a similar diffractogram either at room temperature or in a climatic chamber. A slight shift in melting point was found when comparing the formulation using Florite as a drying agent with the formulation without it.

### XRD analysis of granules

XRD analysis was used to identify the crystalline phases within the material ^43,44^. Based on the x-ray diffraction patterns of mineral granules with various fillers andFlorite as a drying agent, it was evident that the peak intensities differed among the samples. Lactose anh. granules and lactose anh. granules withFlorite 10% exhibited sharp and high diffraction peaks due to the crystalline properties of lactose anh. as a filler. In contrast, Avicel granules, Avicel granules withFlorite 10% addition, as well as Avicel powder displayed less pronounced crystalline patterns (Figs. 5 and 6b). The diffraction pattern of Avicel was characterized by its microcrystalline structure, resulting in broad peaks due to its low crystallinity^45^. Notably, the addition ofFlorite 10% to the mineral granules did not alter the crystalline form of the filler, as exhibited by lactose anh. (Fig. 6a). Thus, the granules failed in the stability study, which was performed after 5 months of storage. These results are in line with previous data on LoD and DSC thermograms (Figs. 2 and 4). In conclusion, a formulation of moisture-resistant granules containing deep seawater, which is physically stable, was successfully developed using Avicel PH 102 as a filler by adding a desiccant/drying agent.

### Study on morphology and surface particles by using scanning electron microscopy (SEM)

SEM analysis was conducted to characterize the morphology and structure of the deep seawater granules. SEM also aids in determining the structure and homogeneity of particles^46^. Based on the morphological observations of granules with Avicel as filler and Avicel granules with Florite 10% addition, it was found that Avicel granules with the addition of Florite exhibited a more porous morphology compared to other formulations (Fig. 7), resulting in increased LoD (Fig. 2a) as well as higher melting points in the Avicel or lactose anh. filler formulations (Fig. 3).

The Morphological analysis of granules with lactose anh. as filler revealed that the granules with Florite 10% addition were physically improved in morphology compared with those without drying agent. Lactose anh. powder typically appears as heterogeneous flakes, whereas on the day of formulation, granules with lactose anh. filler exhibit a dense, non-porous texture. A few hours after granulation, a stability problem arose, and the granules became hygroscopic (G-lact, Fig. 8), leading to the formation of melted particles. With the addition of Florite as a drying agent, the granules became more stable, showing a porous particle morphology. In terms of hygroscopicity, porous morphology tends to be more adsorptive because of its larger surface area for interaction with surrounding molecules. Materials with porous morphology can absorb and retain moisture more efficiently^47^. Nevertheless, the adsorptive property of the drying agent at 10% concentration was not strong enough to stabilize the microscopic nature of lactose anh., thus these formulations failed in stability studies, which were performed for 5 months of storage. Based on stability testing, the findings of this study showed that deep seawater concentrate can be produced into granules by employing Avicel as a filler (Fig. 9). The addition of Florite 10% as a drying agent was the most favorable outcome because of more individualized particles after the study, either at room temperature or climatic chamber (Fig. 9).

### Antistunting activity study

The anti-stunting activity of deep-seawater concentrate samples before and after encapsulation into granules (G-Lact + Fl) was evaluated in vivo based on three parameters: hatching rate, brain length, body length and cartilage growth of zebrafish embryos. The sample treatment on the hatching rate was aimed to determine the hatching rate of the embryos compared to a normal zebrafish control group which was 48–72 hpf or 2–3 dpf^48^. A faster hatching rate is the result of stimulation on enzyme activity, thus an increase in embryo movements^49^. Accordingly, embryo movement, which defines the muscle strength of the larva, is highly affected by nutrients provided by the media environtment. A sufficient nutrient intake that penetrates the corion of the larva will strengthen its muscle and, in the end, will support spontaneous movement for egg hatching. As expected, the group which showed the highest (99.33%) and fastest hatching rate at 3 dpf was the Control Egg Water group (Fig. 10). The group of Controlled eggs treated with rotenone (12.5 ppb) as a Stunting Inducer agent showed delayed hatching (56,67%). rotenone was used as a negative control, namely the group experiencing stunted growth due to its ability to inhibit mitochondria I, thus a decrease in ATP production,^50,51^ followed by high ROS (reactive oxygen species) causing cytochrome C release and Caspase 3 activation resulted increase in apoptosis^52^. ATP as a processed product from Glucose to pyruvate plays the main role in Glucose transport into the cell as GLUT 1 ^53^. A decrease in ATP will highly influence the formation of muscle cells, bone (Osteocalcin as osteoblast product), as well as neuron cells. Disturbance in muscle cell growth inside the larva due to stunted conditions has been quantitatively proven by a lower hatching rate (Fig. 10). At 3 dpf investigation, treatment with deep seawater concentrate after being induced into stunted conditions by rotenone has increased the hatching rate (80%) compared with the stunted group without treatment (56.67%). Surprisingly, the group treated with granules of deep seawater concentrate after rotenone induction showed the highest hatching rate (86.66%), which may due to additional nutrition of sugar derivate (lactose anh.) to the larvae as a granule filler. It can be concluded that treatment with deep-seawater concentrates can increase the hatching rate of stunted zebrafish larvae.

Malnutrition of neurotic cells leads to growth disturbances such as stunted conditions (Muscular Dystrophy Association, 2016). The main effect of stunting on neurocognitive growth is in the dwarf brain,^3^ with a healthy and optimum size depicting normal embryo growth. It is very important to define the activity and connectivity pattern between distinct units within the nervous system^25^. The brain size in zebrafish embryos is measured as its length, which is 1.5 mm at the age of 5 dpf. The brain consists of neurons, and the size will grow with age, reaching circa 4.5 mm long upon reaching adulthood^54^. Thus, the effect of sample treatment on the brain length of zebrafish is an important parameter in antistunting activity due to the effect of stunting on neurocognitive growth^3^.

As expected, the smallest zebrafish brain was found in the group treated with rotenone (1.050 mm), a stunting inducer agent (Fig. 12). Accordingly, the group treated with deep seawater granules after rotenone induction had the longest brain (1.117 mm), while the egg water in the control group was 1.116 mm. This can also be explained by the presence of sugar (lactose anh.) as a filler in the granule formulation, which provides nutrition to the embryo. The group of embryos treated with deep seawater following rotenone treatment showed improved brain growth (1.108 mm) compared to that without treatment after rotenone induction (1.050 mm). Based on these results, it can be concluded that, by evaluating the parameters of brain growth (measured as length), the stunting condition caused by rotenone induction in zebrafish can be improved by treatment with deep seawater because of its rich mineral content.

According to the World Health Organization (WHO) definition, stunting is the impaired growth and development that children experience from poor nutrition, repeated infections, and inadequate psychosocial stimulation. Children are defined as stunted when they show a decrease in body length compared with the normal standard in their growth^24^. Therefore, in this experiment, the body length of zebrafish as an animal model of antistunting activity is an important parameter to be studied. rotenone, which was subjected to the zebrafish embryo, significantly decreased larval growth and body length compared with the other animal groups (Figs. 13 and 14). This result is in line with a previous study that found that rotenone (12.5 ppb) decreased the body length of zebrafish larvae^24,25^. Similar to observations on hatching rate and brain length, the antistunting activity measured by the parameter of body length showed that introduction of deep seawater either as concentrate or as granules had improved the larval growth that had been induced to experience stunting conditions by rotenone.

Stunting directly affect bone growth or development. Pabic et al. (2022) found that cartilage growth in zebra fish has similarities to bones in humans so that understanding the mechanism of human’s bone growth with stunting conditions can be studied with the stunted zebra fish as animal model ^55^. Figure 15 represents ceratohyal (CH) angle of embryos after treatments including control egg water (K), control rotenone (R), emrbryos induces by rotenone followed by sample of deep seawater (R + M), and those followed by granule of deep seawater (R + G). Based on the results of angle measurement from cranial cartilage it is found that angles of ceratohyal (CH) Meckel (M), palatoquadrate and Meckel cartilage (PQ-M), as well as palatoquadrate and ceratohyal view (PQ-CH) after the treatments by rotenone; deep seawater; and the deep seawater granule was significantly affected compared with that by control egg water at *p* < 0.05. The groups treated by rotenone continued by deep seawater (R + M) and that treated by rotenone continued by the granule (R + G) showed the cranial angles close to that of egg water control group (Fig. 16). This can be explained by the improvement in cranial cartilage development due to mineral supplementation from deepseawater and its granule.

Cartilage development is a dynamic process governed by the critical time of differentiation, proliferation, migration, and remodeling of the extracellular matrix of cranial nerve chrysta cells, involving a number of growth factors and enzymes^56^. Bone growth and regeneration are related to four important properties in bone regeneration. First, the osteoconductive matrix acts as a scaffolding or skeleton where bone growth occurs. Second, osteoconductive factors stimulate bone growth, including growth factors, such as bone morphogenetic proteins and growth-β (TGF-β) transformations. Third, osteogenic cells include osteoblasts, primitive mesenchymal cells, and osteocytes, which guarantee structural integrity^57^. This is greatly influenced by nutrient intake derived from nutritious foods thus food deficiencies will directly impact the bone growth.

## Methods

### Chemical content analysis of deep seawater concentrates

If the chemical contents of the deep-seawater samples were analyzed using X-Ray Fluorescence (XRF). The tests were conducted by adjusting the helium gas at a flow rate of 0.66 L/minute with a pressure of 10–15 psi. Before analysis, the XRF instrument was calibrated using an “MCA calibration sample” ^58^.

The chemical contents of the samples were analyzed by ICP-OES (Inductively Coupled Plasma–Optical Emission Spectrometry). Calibration was performed using preprepared calibration solutions. The samples were destroyed beforehand owing to their propensity for precipitation and instrument sensitivity. Standard solutions were prepared for each element. Following the completion of testing with ICP-OES, the results from both XRF and ICP-OES analyses were compared^59^.

### Production of deep seawater granules

The granules were formulated using two types of fillers, lactose anh. and microcrystalline cellulose (Avicel PH 102), using Florite as a dessicant/drying agent. The formulas for the granules are listed in Table 2. The granules were prepared in a fluid bed dryer by spraying deep seawater concentrate containing amylum 10% as a binder into the filler powder. After finishing the spraying process, the granules were dried for 15 min, and Florite was added as a desiccant and prepared for further processing, including physical evaluation and stability study.

**Table 2:** Formula for deep seawater granules.

No.	Formula	Filler	Desiccant	Binder (%)
1	G-AV	Avicel PH 102	–	Amylum 10
2	G-Av- Fl	Avicel PH 102	Florite	Amylum 10
3	G-Lact	Lactose anh.	–	Amylum 10
4	G-Lact-Fl	Lactose anh.	Florite	Amylum 10

### Evaluation of granules

- Organoleptic Evaluation.
  The mineral granules underwent organoleptic evaluation, including assessments of color and shape.
- Flow Properties Evaluation.
  Granule as much as 25 g were passed through a flow tester, and the time taken for the sample to flow was recorded. A good flow rate is generally defined as more than 10 g/s ^60,61^.
- Angle of Repose.
  Granule samples were carefully poured into a flow tester. Upon opening the bottom part, the diameter (*d*) and height (*h*) of the powder heap were measured and calculated using the following formula. A smaller angle indicates better flow properties, while larger one suggests poor flowability. A good flow property is indicated by an angle of repose of less than 35o^60,61^.
  $$\:{tan}^{-1}\:=\frac{2\left(h\right)\:}{\left(d\right)}$$
- Compressibility/Carr’s Index.
  Compressibility characterizes the material’s capacity to decrease in volume under pressure, with values under 15% indicating good flowability and that over 25% indicating poor flow flowability. The compressibility factor was calculated using the following formula ^60,62^. V_0_ is the unsettled apparent volume, and V_f_ is the final tapped volume.
  $$\:Compressibility\:\%=\frac{{V}_{0}\:-\:{V}_{f}}{{V}_{0}}\times\:100$$
- Loss on Drying (LoD).
  One gram of the granules was placed in a moisture-balance apparatus. The apparatus was set to 105 °C until a constant weight was obtained. The results were observed on the screen upon completion. Regular checks were conducted every month for 5 months ^63,64^.
- Stability Testing.
  The Physical stability tests of the granules were carried out for 5 months using climatic chamber (40 ± 2 °C, RH 75 ± 5%) and at room temperature (25 ± 2 °C, RH 70 ± 5%). To evaluate the moisture protection ability of the system, physical stability testing of the granules was performed using organoleptic, LoD, Differential Scanning Calorimetry (DSC), XRD, and SEM^65^.
- Quality Parameter Testing of Granules Using Instruments.
  Advanced characterization of the granules was conducted by evaluating their thermal behavior using DSC, crystallinity by XRD, and morphology by SEM. In this study, DSC testing (Shimadzu 60 A plus) was conducted by placing the sample (5–10 mg) into a crimping (sample) pan, and the nitrogen and oxygen gas bases were turned on. The difference in heat flow between the sample and reference was noted as the temperature changed. The resulting data were plotted as a function of the temperature, resulting in a DSC curve^66^.
  Powder X-ray diffraction (XRD, Malvern Panalytical) patterns of the granules were recorded using a Cu anode X-ray tube, and the generator voltage and current were 40 kV and 30 mA, angle of 2θ in increments of 2 °C/min^67^.
  SEM (Scanning Electron Microscopy) study of the granule used an electron microscope (JED-2300 Analysis Station) for viewing the morphology of the surface of the granules. The working principle of SEM is that the sample is placed at the bottom of the electron column. The electrons in the sample were scattered and captured by an electron detector. Photomultipliers converted voltage signals into images on a PC screen. The magnifications used in this test were 200x, 1000x and 10,000 × ^68^.

### Antistunting activity

#### Preparation of solutions

An egg water solution was prepared by mixing 1 L Reverse osmosis (RO) water with methylene blue and 1.5 ml salt stock^69^. Salt stock was prepared by mixing 40 g fish salt with 1 L RO water. rotenone solution was prepared by dissolving rotenone (Sigma, R8875) impurity > 95% in egg water and diluting to 12.5 ppb. This concentration has also been shown to inhibit the zebrafish growth^24,25^.

### Animals and experimental design

Wild type zebrafish (*Danio rerio*) were obtained from Leiden University and cultivated at the Faculty of Biology, Gadjah Mada University. The fish were maintained in a cycled aquarium at room temperature between 27 and 28 °C and feed three times a day. The embryos used in this experiment were aged 2 Hpf (hours post-fertilization) which were observed until 9 dpf (days post-fertilization). They were fertilized from one male and two adult female zebrafish on media at 27–28 °C.

The experimental design consisted of four groups: (1) control embryos in egg water media; (2) embryos treated with rotenone (12.5 ppb); (3) embryos treated with rotenone (12.5 ppb), followed by deep seawater granules with magnesium content equivalent to 0.05238 ppt; and (4) embryos treated with rotenone (12.5 ppb), followed by deep seawater at 84.73 × 10⁻⁸ µl, also equivalent to magnesium 0.05238 ppt. Each group was replicated three times, with each replicate consisting of 10 embryos, resulting in 30 embryos per group ^70^.

Magnesium, one of the main minerals found in deep seawater, was subjected to the zebrafish embryos based on dose conversion for embryos compared with adult human’s dose of magnesium. The concentration of 0.05238 ppt magnesium was chosen based on the calculated nutrient dose for zebrafish embryos, which have an average weight of 0.95 mg. This magnesium dose was then applied to the treatment groups as described above.

All experimental procedures were conducted in accordance with ethical standards established by the Research Ethics Committee of Universitas Padjadjaran, Bandung, with Ethical approval from the Research Ethics Committee at Universitas Padjadjaran Bandung under Ethical Approval No. 189/UN6.KEP/EC/2025. All methods were carried out in accordance with relevant guidelines and regulations, and the study is reported in compliance with the ARRIVE guidelines (https://arriveguidelines.org).

### Stunting parameters evaluation

Several parameters for stunting evaluation, including hatching rate, brain length, and body length, were measured to living zebrafish. For cranial cartilage evaluation, zebrafish were euthanized using the rapid chilling method in accordance with the American Veterinary Medical Association (AVMA) Guidelines on Euthanasia: 2020 Edition.

#### Hatching rate

Embryos were observed at 3–5 dpf Normally, embryos hatch at 72 hpf or 3 dpf, and observations until 5 dpf are conducted to evaluate the delay of egg hatching.

#### Brain length

Brain length observation was conducted by measuring the length of the embryo’s brain at 24 hpf, when brain division can easily be seen in a microscope (Leica ICC50E).

#### Body length

The length of embryos was measured at 3, 6, and 9 dpf, the larva on the object glass was observed using a microscope, and images were taken to measure body length using ImageJ software from the anterior nose (tip of the snout) to the end of the tail fin (caudalfin) or snout–fin^69^.

**Fig. 17:**
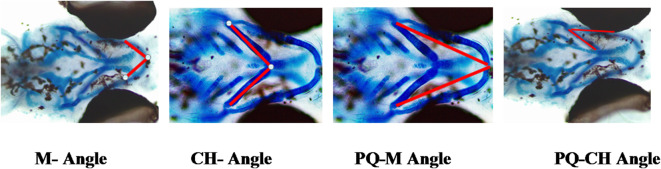
Cranial morphology of Zebra fish’s head cartilage after ARAB staining from different view of angles.

**Cranial Cartilage morphology**: Embryonic Cartilage development were conducted by observing ceratohyal (CH) parameter, Meckel angle (M), palatoquadrate and Meckel cartilage angle (PQ-M), as well as palatoquadrate and ceratohyal angle (PQ-CH). The morphologies were observed based on staining reaction using ARAB (Alizarin Red Alcian Blue). The larve at age of 9 Dpf were euthanized by using rapid chilling in accordance with the AVMA Guidelines on Euthanasia: 2020 Edition. Following euthanasia, the specimens were fixed in 96% ethanol, then soaked in ARAB for three days for staining. After staining, the samples were washed thoroughly with distilled water, followed by bleaching in a 0.05% KOH solution for 24–36 h. Next, the solution was replaced by Moll’s solution and after 14 h it was removed and replaced by Glycerin for storage. The samples were observed under Leica ICC50E Microscope at 100x magnification. Cartilage development were observed by viewing parameters angles of ceratohyal (CH), Meckel (M), palatoquadrate and Meckel cartilage (PQ-M), as well as palatoquadrate and ceratohyal (PQ-CH) (Fig. 17).

## Conclusions

Deep seawater concentrate contains magnesium (Mg), chloride (Cl), potassium (K), calcium (Ca), iron (Fe), copper (Cu), and sodium (Na), and is a potential source of minerals for multivitamin. Despite very hygroscopic in nature, the formulation of deep seawater into granules is highly needed due to practical purposes and it had been successfully conducted. It can be concluded that Avicel PH 102 (as a derivative of microcrystalline cellulose) was the most promising filler to provide good properties and stability of the granules in terms of moisture protection of highly hygroscopic granules. The addition of Florite as dessicant into the powder improves the physical properties of the granules. An anti-stunting activity study on deep-sea water concentrate and granules showed potential results and revealed that they can improve zebrafish growth by increasing hatching rate, brain length, and body length after stunting induction by rotenone. The cranial cartilage development of the fish after stunting inducement were found to be improved after treatment with deep seawater either as concentrate or as granules.

## Data Availability

I state the datasets used and/or analyzed during the current study available from the corresponding author on reasonable request. (corresponding author: [anis.yohana.chaerunisaa@unpad.ac.id] (mailto: anis.yohana.chaerunisaa@unpad.ac.id) )
